# Malnutrition and associated factors in patients with infectious diseases

**DOI:** 10.1186/1471-2334-14-S7-P43

**Published:** 2014-10-15

**Authors:** Maria Nițescu, Adrian Streinu-Cercel, Daniela Pițigoi, Cristina Olariu, Anca Moldoveanu, Dana Galieta Mincă, Florentina Furtunescu

**Affiliations:** 1 Carol Davila University of Medicine and Pharmacy, Bucharest, Romania; 2 National Institute for Infectious Diseases "Prof. Dr. Matei Balş", Bucharest, Romania

## Background

Worldwide, malnutrition affects 20-50% of patients admitted to hospitals. Malnutrition has a high impact in clinical outcomes and cost of healthcare. In Romania, there is a general lack of hospital malnutrition data, and also of malnutrition prevalence in infectious diseases hospitals.

Objective: to assess malnutrition risk and associated factors in hospitalized patients with infectious diseases.

## Methods

Cross-sectional study that included all patients above 18 years old (207), consecutively admitted in the National Institute for Infectious Diseases "Prof. Dr. Matei Balş", Bucharest, during June 16 – July 16, 2014. Malnutrition risk was assessed by malnutrition universal screening tool (MUST) in the first 24 hours of admission and associated factors were collected from the clinical observation file. We compared the patients with high malnutrition risk (MUST ≥2) with patients without risk or moderate risk (MUST 0 and 1), regarding demographical features (gender, age) and severity of clinical status (number of pills, length of stay, number of previous hospitalizations). The quantitative variables were analyzed as central tendency and dispersion, and qualitative variables were analyzed as proportions. For comparison, we used the T-student test, Mann Whitney U test and Chi^2^ test, as applicable.

## Results

Malnutrition was diagnosed in 23% of patients according to MUST, 17% and 6% of them having high risk (MUST ≥2) and moderate risk (MUST 1), respectively. With respect to age, we observed a statistically significant difference between patients with high risk of malnutrition and patients with low risk (mean age 60±3.592 years vs. 49.99±19.736 years, p=0.007). Regarding the severity of clinical status, there was a statistically significant difference in the mean number of previous hospitalizations between patients with high risk and patients with low risk (1.81 vs. 1.19, p=0.003). Also, the patients with high risk had an average length of stay higher than patients with low risk (8.31 days vs. 6.91 days), statistically insignificant. The mean number of pills administered per day was higher in patients with high risk compared to patients with low risk (2.56 pills vs. 1.91 pills), statistically insignificant. 8.3% of patients with high risk of malnutrition and 6.4% of patients with low risk received more than 5 pills/day, statistically insignificant.

## Conclusion

In our study, high risk of malnutrition was significantly associated with older age and number of previous hospitalizations. We need a larger study group to emphasize other variables significantly associated with high risk of malnutrition.

